# The precision by the Face Arm Speech Time (FAST) algorithm in stroke capture, sex and age differences: a stroke registry study

**DOI:** 10.1136/bmjno-2023-000574

**Published:** 2024-04-15

**Authors:** Guri Hagberg, Haakon Ihle-Hansen, Tamar Abzhandadze, Malin Reinholdsson, Adam Viktorisson, Hege Ihle-Hansen, Katharina Stibrant Sunnerhagen

**Affiliations:** 1 Department of Clinical Neuroscience, Institute of Neuroscience and Physiology, Sahlgrenska Academy, University of Gothenburg, Goteborg, Sweden; 2 Oslo Stroke Unit, Department of Neurology, Oslo University Hospital, Oslo, Norway; 3 Department of Medicine, Vestre Viken Hospital Trust, Drammen, Norway; 4 Department of Occupational Therapy and Physiotherapy, Sahlgrenska University Hospital, Goteborg, Sweden; 5 Department of Neurology, Oslo University Hospital, Oslo, Norway; 6 Neurocare, Sahlgrenska University Hospital, Goteborg, Sweden

**Keywords:** STROKE

## Abstract

**Background:**

The shift towards milder strokes and studies suggesting that stroke symptoms vary by age and sex may challenge the Face-Arm-Speech Time (FAST) coverage. We aimed to study the proportion of stroke cases admitted with FAST symptoms, sex and age differences in FAST presentation and explore any additional advantage of including new item(s) from the National Institute of Health Stroke Scale (NIHSS) to the FAST algorithm.

**Methods:**

This registry-based study included patients admitted with acute stroke to Sahlgrenska University Hospital (November 2014 to June 2019) with NIHSS items at admission. FAST symptoms were extracted from the NIHSS at admission, and sex and age differences were explored using descriptive statistics.

**Results:**

Of 5022 patients, 46% were women. Median NIHSS at admission for women was (2 (8–0) and for men 2 (7–0)). In total, 2972 (59%) had at least one FAST symptom, with no sex difference (p=0.22). No sex or age differences were found in FAST coverage when stratifying for stroke severity. 52% suffered mild strokes, whereas 30% had FAST symptoms. The most frequent focal NIHSS items not included in FAST were sensory (29%) and visual field (25%) and adding these or both in modified FAST algorithms led to a slight increase in strokes captured by the algorithms (59%–67%), without providing enhanced prognostic information.

**Conclusions:**

60% had at least one FAST symptom at admission, only 30% in mild strokes, with no sex or age difference. Adding new items from the NIHSS to the FAST algorithm led only to a slight increase in strokes captured.

**WHAT IS ALREADY KNOWN ON THIS TOPIC**

- The FAST (Face, Arm, Speech, Time to call) algorithm is a widely used public campaign, historically reported to cover three out of four patients who had a stroke. The shift towards milder strokes and research indicating sex and age differences in symptom presentation challenge this knowledge.

**WHAT THIS STUDY ADDS**

- The precision by the FAST algorithm is low in mild strokes, without any sex or age differences.

**HOW THIS STUDY MIGHT AFFECT RESEARCH, PRACTICE OR POLICY**

- Healthcare personnel and the public need to be aware of the low FAST algorithm precision and emphasise the importance of potential stroke symptoms knowledge and public education.

## Introduction

 The Face, Arm, Speech, Time to call (FAST) is a widely used algorithm in public campaign and emergency medical services to improve symptom recognition in acute stroke^1^ and was initially found to capture up to 90% of ischaemic strokes and transient ischaemic attacks (TIAs).^2^ It has formed the basis of public awareness and education in many countries. The FAST algorithm has improved both symptom recognition and intention to call emergency medical services after major stroke,^3^,^5^ but with less precision in minor strokes or TIA.^6^ Recent stroke register data show a shift towards less severe strokes with an increased incidence of stroke in young adults, and the precision by the FAST acronym in capturing stroke seems lower than previously reported.^6^,^8^

In general, women have worse outcomes after stroke compared with men,^9^ partly explained by prestroke disability, higher age and larger strokes due to higher occurrence of atrial fibrillation.^10 11^ However, recent research indicates sex differences also in the presentation and management of acute stroke. A large population-based study identified sex differences in prehospital management by emergency medical services,^12^ and women may be more likely to receive the diagnosis of stroke mimics.^13^ Two recent large systematic reviews and meta-analyses reported sex differences in the occurrence of non-focal symptoms, such as level of consciousness and headache, while evidence for sex differences in focal symptoms remains uncertain.^14 15^ Atypical clinical presentation is more common in younger ages, followed by a larger portion of missed stroke diagnoses in the emergency department (ED).^16^ Misdiagnosis in the acute phase and consequently missed treatment can lead to disability. Therefore, healthcare personnel and the public need increased awareness of any sex or age differences in the FAST precision.

The National Institute of Health Stroke Scale (NIHSS) quantifies stroke severity and is extensively used in acute stroke care worldwide.^17 18^ The 15-item scale covers all symptoms included in FAST; facial palsy, arm weakness and speech (dysarthria of aphasia), besides measures of motor leg, sensory function, ataxia, visual field, level of consciousness, attention and neglect. Studies show that vision impairments are common and reduce the odds of poststroke independence.^19 20^ Ideally, including vision known to impact prognosis or other frequently presented stroke symptoms in the FAST algorithm could increase the number of strokes captured by the algorithm and provide enhanced prognostic information. Further, more identified stroke cases could result in a higher proportion of acute treatment and reduce the burden of stroke-related disability. Overall, public campaigns face the balance between an easily remembered acronym and the often more complex reality.

Using data from Swedish stroke registers, we aimed to study the proportion of stroke cases admitted with FAST symptoms, sex and age differences in FAST presentation, and explore any additional advantage of including new item(s) from the NIHSS to the FAST algorithm.

## Methods

### Study design

This registry-based study used data from two Swedish stroke registers (Riksstroke, the mandatory national quality register for stroke care in Sweden, established in 1994^21^ and Väststroke, a local quality stroke register in Gothenburg, collecting data from 2014 to 2019). The two registers provide different information about the same patients. Dedicated nurses at the stroke units are responsible for reporting data to both registers on patients. The datasets were merged by a statistician at Riksstroke through personal identification numbers into one pseudonymised database.

### Study sample

All patients admitted with an acute stroke to the Sahlgrenska University Hospital from 1 November 2014 until 31 June 2019 were included. The Sahlgrenska University Hospital consists of three sites, each with a comprehensive stroke unit. It provides emergency and basic care for 800 000 inhabitants in Gothenburg as well as specialised care for 1.8 million inhabitants in the west of Sweden. Patients matching the inclusion criteria were adults (≥ 18 years) with the diagnosis of ischaemic stroke (I63) or intracerebral haemorrhage (I61) according to the International Classification of Diseases 10th Revision and with complete NIHSS at admission.

### Procedure and study variables

The NIHSS with scores on all items at admission is available in Väststroke and administered by physicians. When possible, assessments were retrieved from medical records for cases with missing NIHSS in the Väststroke register by dedicated nurses.^22^ The follow-up data on functional outcomes were obtained from the Riksstroke 3-month follow-up questionnaire.^23^ The hospital administered the questionnaire and posted it to all registered patients who had a stroke 3 months earlier. The questionnaires could be completed by the patient or a relative or caregiver if the patient could not.

The NIHSS quantifies stroke severity^17 18^; the score range is 0–42, where 0 indicates no neurological deficits. In descriptive statistics, total score and stratified NIHSS scores were used. The stratification was as follows: mild stroke (NIHSS ≤3), mild to moderate stroke (NIHSS 4–15) and severe stroke (NIHSS ≥15). FAST symptoms were extracted from the NIHSS at admission. Patients were dichotomised into FAST negative (no FAST symptom (score 0)) or FAST positive (symptom(s) (score of 1 or more)). FAST positive includes patients with one or more of the NIHSS items: facial palsy, motor arm, best language and dysarthria.

For the explorative regression analyses, we restricted our analysis to patients with both NIHSS scores at admission and calculated Modified Rankin Scale (mRS) values at 3 months. The primary explanatory variable was the presence of FAST symptoms (FAST positive) at admission, and the outcome variable was functional status at 3 months after stroke, calculated using an algorithm transforming the self-reported outcomes collected by the questionnaire at 3 months follow-up visit or telephone interview to mRS.^23^ The algorithm cannot discriminate mRS 0, 1 and 2. Therefore, we have used dichotomised scores, where mRS ≤2 indicated functional independence, and mRS >2 indicated functional dependency. We also wanted to analyse modified FAST algorithms, including the two most common focal NIHSS items, separately and together.

Other variables used in the study were sex, stroke type (I61 and I63), premorbid dependency, premorbid cardiovascular risk factors and comorbidities. The death date was retrieved from Statistics Sweden.

### Statistics

We present the results stratified for sex and stroke severity. Continuous variables are given as mean±SD or as median and IQR as appropriate, and categorical variables are presented as number and percentage (%). Mann-Whitney U-test was used for comparison of non-normally distributed continuous variables, and the χ^2^ test for categorical variables. We examined the occurrence of NIHSS item symptoms that are not included in the FAST algorithm, and the two most common focal symptoms were added to FAST in various combinations to investigate the added value of a modified FAST algorithm.

Regression analysis included only patients with available transformed mRS at 3 months follow-up and complete NIHSS at admission. Binary logistic regression analyses were carried out, unadjusted and adjusted for sex (male, female), age and stroke type (haemorrhage, ischaemic) to explore an association of FAST and modified FAST algorithms on functional dependency (mRS >2) 3 months after stroke. Prior to regression analyses, the observations with missing values were omitted.

The regression analyses were performed as follows: the data on patients who had a stroke concerning the period from November 2014 to 31 May 2018, n=2251, were randomly divided into training (80%, n=1802) and test sets (20%, n=449). The data on patients who had a stroke concerning the period from June 2018 to 1 June 2019 (n=283) were used as a validation set. Each model was fitted using the training data. ORs with 95% CIs and p values were obtained for each variable. The model developed on the training data was evaluated using the test data set. For model evaluation, the area under the curve (AUC) and 95% CIs for AUC were used. The model performance and stability were assessed by fitting the regression model in the validation set. AUC values with 95% CIs were evaluated, with same or higher AUC values indicating a good fit for the model. All statistical tests were two-tailed at alpha 5%. The analyses were performed in RStudio, V.2022.7.1.554 (regression analyses) and IBM SPSS Statistics (V.28).

## Results

**Figure 1:**
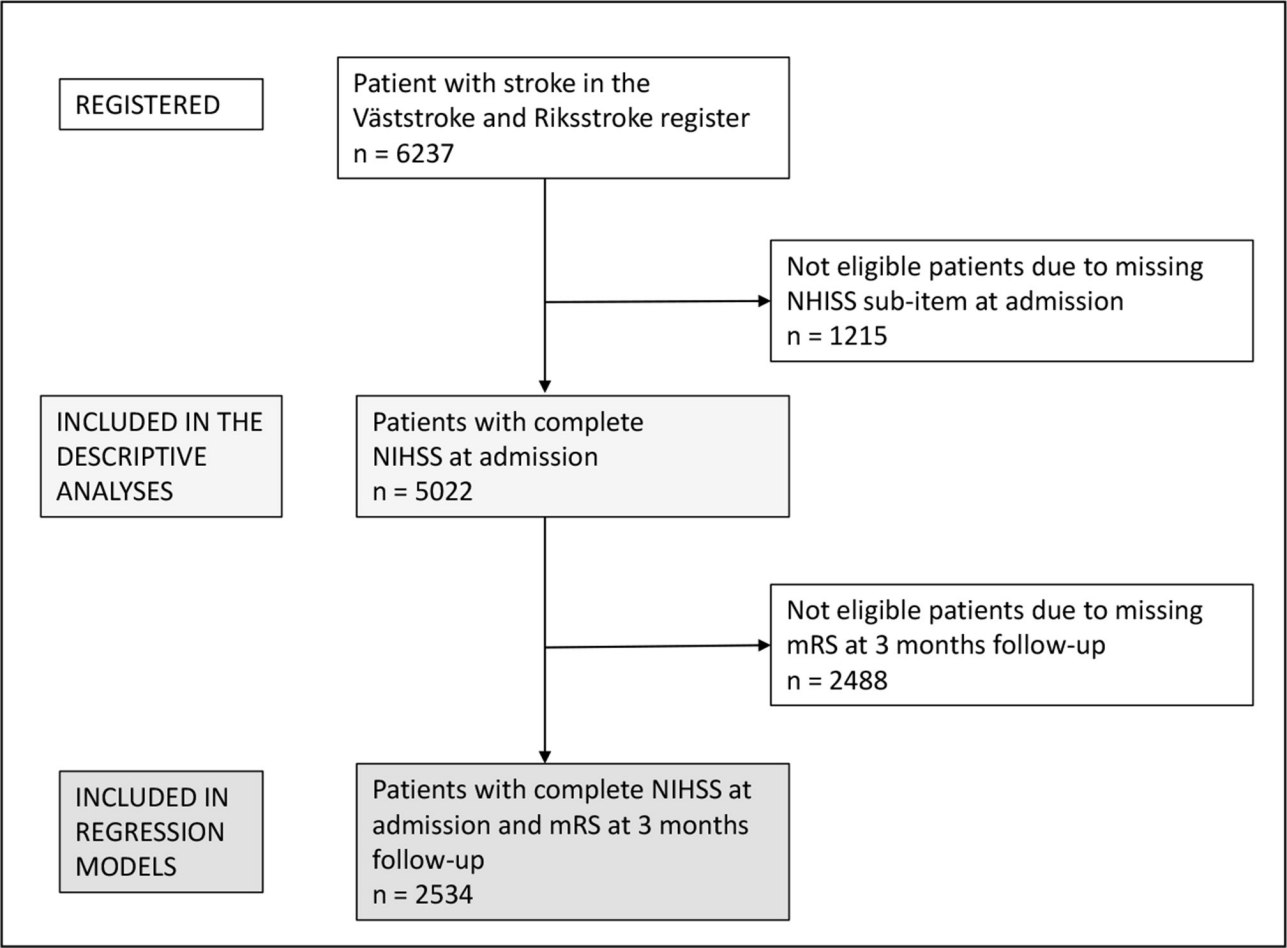
Complete NIHSS=available scores on at least one of the NIHSS subitems consciousness, orientation and/or motor arm at admission. NIHSS, National Institute of Health Stroke Scale. Modified Rankin Scale (mRS)

Out of 6363 patients admitted with acute stroke, 5022 (80.5%) had complete NIHSS at admission and were included in this study (flow chart, figure 1). Patients not included due to missing NIHSS at admission were older, with more severe strokes, and 430 (32%) died before the 3-month follow-up (online supplemental table 1).

Of the 5022 patients included in this study, 2312 (46%) were women. Women were older, with a mean (SD) age of 76.0 (13.9) years, compared with 71.3 (12.9) in men. In total, 4468 (89%) suffered ischaemic stroke without sex differences. Women had a significantly higher NIHSS than men (mean (SD) NIHSS 6 (7) vs NIHSS 5 (6), p<0.001), with 1127 (56%) and 1460 (60%) of strokes categorised as mild (NIHSS ≤3) for women and men, respectively. Dysarthria (present in 38%), followed by facial palsy (present in 36%), was the most common NIHSS item affected. When combining left and right weakness, weakness in one arm or leg (present in 34%) was the third most common symptom. There was a significantly higher proportion of women with scores on 9 of the 15 NIHSS items (consciousness, orientation, commands, gaze, left arm, motor leg, sensory, best language and neglect). Patient characteristics and sex differences are presented in table 1.

**Table 1:** Characteristics of the study population by sex

	Total 5022	Men 2710 (54.0)	Women 2312 (46.0)	P value
Age (years)				<0.01
Mean (±SD)	73.6 (13.6)	71.3 (12.9)	76.0 (13.9)	
Median (IQR)	75 (84–66)	73 (81–63)	79 (86–70)	
Age groups (years), n (%)				<0.01
18–44	171 (3.4)	85 (3.1)	86 (3.7)	
45–64	962 (19.2)	661 (24.4)	310 (13.4)	
65–79	2005 (39.9)	1174 (43.3)	830 (36.9)	
>79	1884 (37.5)	790 (29.2)	1094 (47.3)	
Stroke type (%)				0.76
Ischaemic	4585 (91.2)	2406 (88.7)	2062 (89.1)	
Haemorrhagic	432 (8.6)	236 (8.7)	196 (8.7)	
NIHSS^1^				<0.001
Mean (±SD)	5 (7)	5 (6)	6 (7)	
Median (IQR)	2 (8–0)	2 (7–0)	3 (9–0)	
Stroke severity (%)^2^				0.003
Mild (NIHSS ≤3)	2587 (51.5)	1460 (60.2)	1127 (56.1)	
Mild to moderate	1364 (27.2)	732 (30.2)	632 (31.5)	
Severe (NIHSS >15)	480 (9.6)	232 (9.6)	248 (12.5)	
Died before 3 months follow-up	538 (10.7)	248 (9.2)	290 (12.5)	<0.01
Premorbid independency (%)^3^	3700 (73.7)	2135 (85.0)	1565 (73.1)	<0.001
NIHSS subitems (%)				
1a Consciousness^4^	473 (9.4)	226 (8.4)	247 (10.8)	0.004
1b Orientation^5^	1454 (29.0)	716 (26.7)	738 (32.5)	<0.001
1c Commands^6^	632 (12.6)	299 (11.2)	333 (14.8)	<0.001
2 Gaze^7^	889 (17.7)	448 (17.0)	441 (25.0)	0.07
3 Visual^8^	1143 (22.8)	601 (23.0)	542 (25.1)	0.101
4 Facial palsy^9^	1739 (34.6)	930 (35.4)	809 (36.4)	0.481
5 Motor arm				
Right^10^	769 (15.3)	395 (14.7)	374 (16.6)	0.074
Left^11^	919 (18.3)	471 (17.6)	448 (19.9)	0.043
6 Motor Leg				
Right^12^	793 (15.8)	385 (14.4)	408 (18.3)	<0.001
Left^13^	931 (18.5)	455 (17.1)	476 (21.2)	<0.001
7 Ataxia^14^	773 (15.4)	416 (16.0)	357 (16.6)	0.749
8 Sensory^15^	1465 (29.2)	764 (29.2)	701 (31.9)	0.04
9 Best language^16^	1465 (29.2)	759 (28.7)	715 (32.6)	0.004
10 Dysarthria^17^	1776 (35.4)	941 (36.2)	835 (38.0)	0.206
11 Neglect^18^	774 (15.4)	385 (15.2)	389 (18.4)	0.003
FAST positive (%)^19^	2972 (59.2)	1588 (59.9)	1384 (61.7)	0.215
Premorbid cardiovascular risk/comorbidity (%)				
Atrial fibrillation^20^	921 (18.3)	472 (17.9)	449 (19.9)	0.074
Diabetes^21^	937 (18.7)	582 (28.5)	355 (5.7)	<0.001
Smoking^22^	610 (12.1)	360 (15.2)	250 (12.7)	<0.021
On statins^23^	1344 (26.8)	774 (29.3)	570 (33.9)	0.002
Hypertension^24^	3032 (60.4)	1578 (59.9)	1454 (64.5)	0.001

Missing data: n: 1591, 2137,3367, 441,580,6103, 7185, 8253, 9171,10101,1193, 12128, 13118, 14243, 15211, 16222, 17228, 18379, 19133, 20133, 21130, 22700, 23138, 24138; FAST positive: scores on at least one of the NIHSS subitems facial palsy, motor arm, best language and dysarthria.

NIHSS, National Institutes of Health Stroke Scale.

### FAST precision by sex, age, and stroke severity

In all, 2972 patients (59.2%) had at least one FAST symptom at hospital admission, and as many women as men were admitted with FAST symptoms (p=0.215). Further, there were no sex or age differences found in FAST precision when stratifying for stroke severity. In mild strokes (NIHSS ≤3), 23%–32% were FAST positive, whereas 94% and above were FAST positive in moderate and severe strokes (table 2).

**Table 2:** FAST symptoms in relation to stroke severity and age by sex

Stroke severity	Age groups (years)	Men positive*/total (%)	Women positive*/total (%)	P value
Mild (NIHSS ≤3)	18–39	18/62 (29)	14/60 (23)	0.474
	40–69	102/401 (25)	53/196 (27)	0.675
	70+	206/649 (32)	127/426 (30)	0.503
Moderate	18–39	18/19 (95)	16/16 (100)	0.352
	40–69	154/159 (97)	64/64 (100)	0.151
	70+	296/306 (98)	210/223 (94)	0.154
Severe (NIHSS >15)	18–39	2/2	4/4	N/A
	40–69	49/49	24/24	N/A
	70+	98/98	88/88	N/A

* Positive: FAST positive: at least one FAST symptom.

NIHSS, National Institutes of Health Stroke Scale.

### NIHSS items and modified FAST algorithms

**Figure 2:**
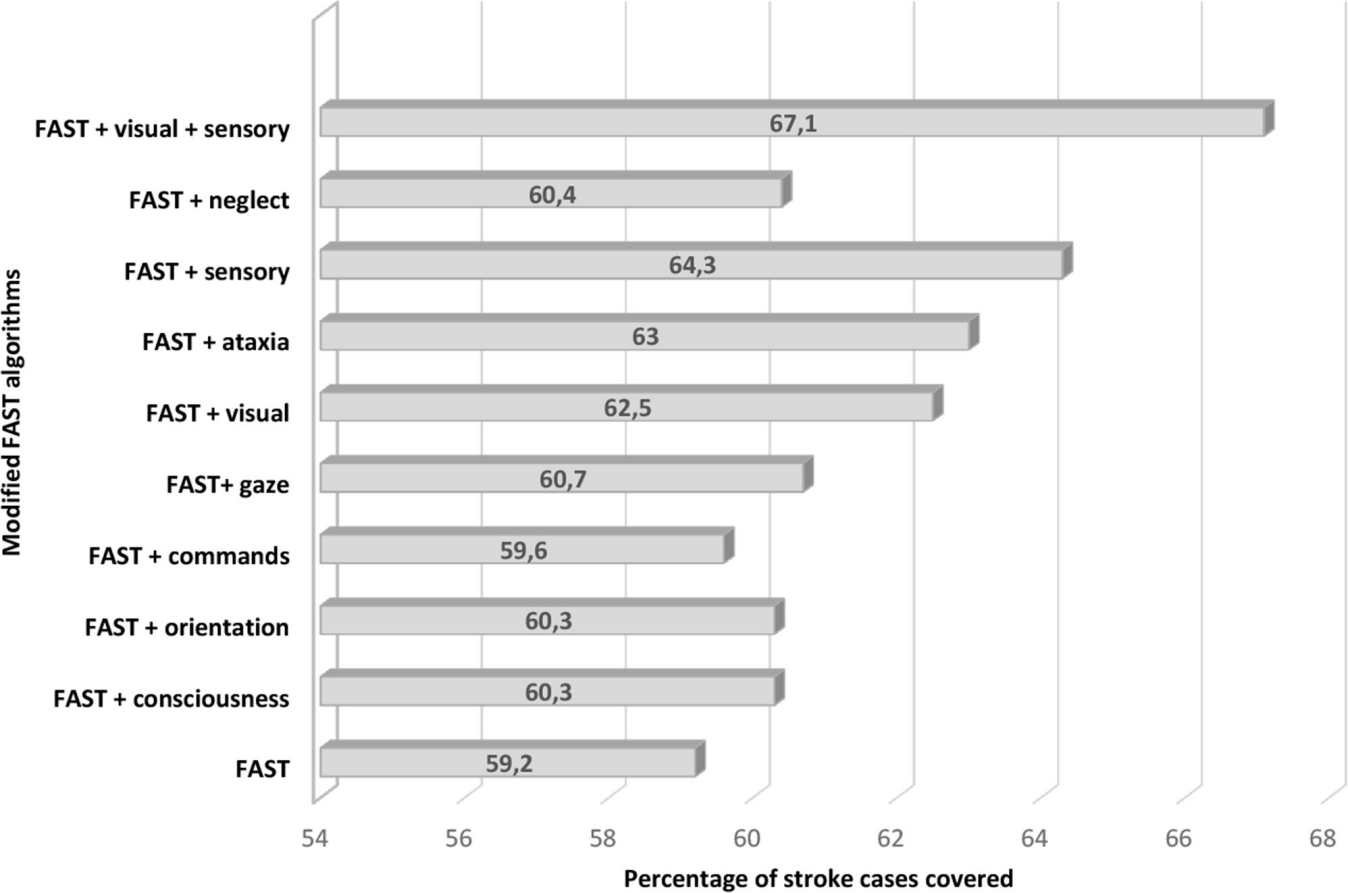
Percentage of 5022 stroke cases covered when including new subitems from the NIHSS to the FAST algorithm. NIHSS, National Institute of Health Stroke Scale.

The most frequent NIHSS items not included in the FAST algorithm were orientation (33%), sensory (29%) and visual field (25%). Orientation is a non-focal symptom, with minimal increase in cases identified when added to the FAST algorithm (figure 2) and was not included in further analysis. Adding the most frequent focal symptoms, sensory and visual field, to FAST, increased the prevalence of at least one symptom from 59% to 67% (figure 2), with no relevant sex differences (online supplemental figure 1). When looking at only minor strokes, covered strokes increased by 12% (from 30% to 42.2%) when adding visual field and sensory to FAST. The most common isolated NIHSS item was sensory (n=127, 2.5%), followed by facial palsy (n=107, 2%). Four patients (0.1%) had isolated scores on consciousness. (Frequencies of isolated NIHSS items by sex are presented in online supplemental table 2.)

**Figure 3:**
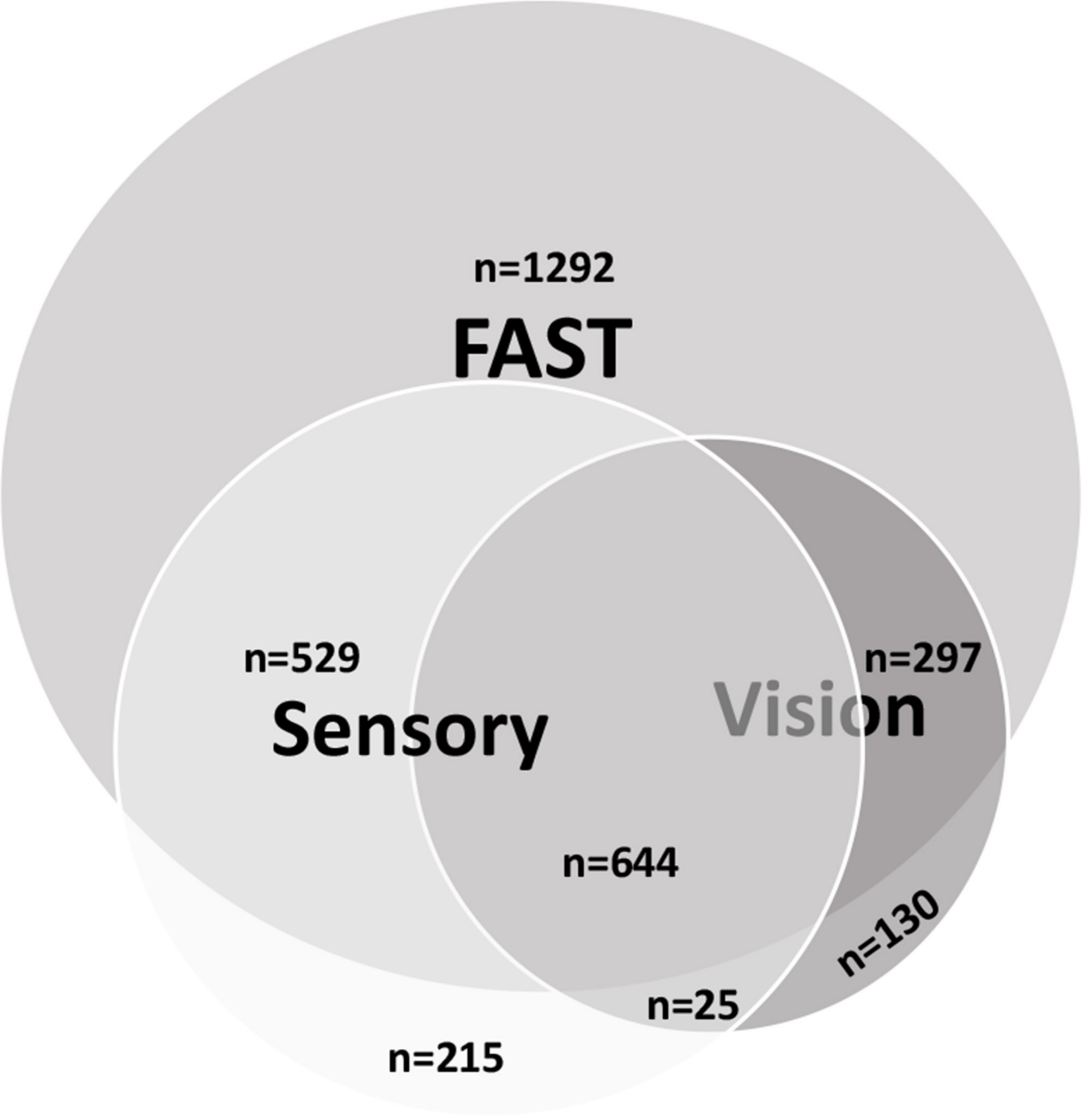
Frequency of FAST symptoms alone or in combination with sensory or visual symptoms. FAST: Face Arm Speak Time.

The frequency of FAST symptoms alone or in combination with sensory or visual field symptoms is shown in figure 3. Only 215 (4%) had isolated sensory symptoms, and 130 (2.6%) had isolated visual field symptoms.

Based on our results, the modified FAST algorithms were FAST-V (visual field); at least one FAST symptom and/or scores on the NIHSS item visual field, FAST-S (sensory); at least one FAST symptom and scores and/or the NIHSS item sensory and FAST-VS; at least one FAST symptom and/or scores on both visual field and sensory symptoms. Slightly more women had FAST-VS (68.3% vs 66.0%), presented in online supplemental table 3.

In the exploratory regression models, only patients with both NIHSS at admission and mRS at 3 months were included (n=2534). The results of the univariable binary logistic regression model showed that FAST and FAST-V algorithms had the highest ORs for explaining dependency (mRS >2) 3 months after stroke (table 3, panel A). Each model could correctly classify 64% of the patients (AUC, 0.64). The AUC values increased by 1% when regression models were fitted in the validation dataset, indicating the stability of model performance. The results of the multivariable binary logistic regression model showed that FAST and FAST-V algorithms had the highest ORs for explaining dependency (mRS >2) 3 months after stroke, ORs 4.56 and 4.54, respectively (table 3, panel B). These models could correctly classify 78%–79% of the patients. When regression models were fitted in the validation dataset, 80% of the patients could be correctly classified, indicating the stability of model performance.

**Table 3:** Univariable and multivariable models with different FAST and subitem combinations for explaining the mRS >2 at 3 months follow-up

	OR (95% CI)	P value	AUC (95% CI), test data	AUC (95% CI), validation data
Panel A: Univariable models				
FAST	4.30 (4.06 to 4.53)	<0.001	0.64 (0.60 to 0.69)	0.65 (0.60 to 0.71)
FAST-V	4.31 (4.07 to 4.56)	<0.001	0.64 (0.59 to 0.68)	0.65 (0.59 to 0.70)
FAST-S	3.56 (3.31 to 3.80)	<0.001	0.63 (0.58 to 0.67)	0.62 (0.57 to 0.67)
FAST-VS	3.65 (3.40 to 3.90)	<0.001	0.62 (0.58 to 0.67)	0.62 (0.57 to 0.67)
Panel B: Multivariable models*				
FAST	4.56 (4.29 to 4.82)	<0.001	0.78 (0.74 to 0.83)	0.80 (0.74 to 0.85)
FAST-V	4.54 (4.27 to 4.81)	<0.001	0.79 (0.74 to 0.83)	0.80 (0.74 to 0.85)
FAST-S	3.90 (3.63 to 4.17)	<0.001	0.78 (0.73 to 0.83)	0.79 (0.73 to 0.85)
FAST-VS	3.94 (3.66 to 4.22)	<0.001	0.79 (0.74 to 0.83)	0.79 (0.73 to 0.85)

OR, 95% CI and p values are given for train dataset. N of observations: training data 1802, test data, 449. Validation data, 283.

* Models are adjusted for age at onset and stroke type.

AUC, area under curve; FAST, Face Arm Speak Time; FAST-S, Face Arm Speak Time Sensory; FAST-V, Face Arm Speak Time Visual field; FAST-VS, Face Arm Speak Time Visual field Sensory; P values, two-tailed p values.

## Discussion

In total, 60% of the 5022 patients included in this study had at least one FAST symptom at admission, but only 30% of those admitted with minor strokes, without any sex or age differences. Including other NIHSS items in the modified FAST algorithms led to a slight increase in strokes captured by the algorithms.

Only 60% had at least one FAST symptom at admission, which is lower than reported in previous studies,^2^ and shows that the general shift towards less severe strokes affects the FAST campaign coverage.^24^ In the current study, 52% had minor strokes, and among them, 30% had at least one FAST symptom at admission. Non-focal and unspecific symptoms like headache, vertigo, unsteady gait and dizziness are common in minor strokes, but often in combination with focal symptoms.^13^ High-quality studies that address different presentations of stroke are lacking. As a result, the absolute increase in stroke cases covered by adding new non-focal symptoms to the FAST algorithm might be limited.^2^ Our stroke register did not report other symptoms than the NIHSS at admission. However, when adding sensory or visual field from the NIHSS to FAST, the coverage increased from 59% to 67%, as only 7% had vision and/or sensory deficits without any other FAST symptoms. When exploring only minor strokes, the absolute increase was similar. The FAST algorithm identifies 95%–100% of all moderate and severe strokes in our register, detecting most cases eligible for thrombectomy with known considerable potential to reduce disability and cost.^25^ Adding common non-focal symptoms, like dizziness and vertigo, to stroke awareness campaigns to capture more minor strokes may lead to an overtriage. Dizziness and vertigo are among the most common causes of ED visits, but in these patients, only 3%–5% have a stroke as the underlying cause.^26^ At the same time, patients presenting with headaches or dizziness are the most misdiagnosed stroke events in the ED,^16^ emphasising their importance.

The FAST algorithm includes the focal symptoms of facial palsy, arm weakness, aphasia/best language and dysarthria. When comparing each item in the FAST algorithm separately, women had more often left arm paresis and aphasia than men. This was also the case for NIHSS in general, as women had larger strokes and more often scores on the NIHSS items consciousness, orientation, commands, gaze, left arm, motor leg, sensory, best language and neglect, comparable to other stroke register data.^27^ We found no sex difference in the presence of at least one FAST symptom. This finding aligns with two large recent systematic reviews and meta-analyses, showing that women had a higher prevalence of some non-focal symptoms at the stroke presentation but no difference in focal symptoms.^14 15^ The two studies do not report if the non-focal symptoms, mental status change, loss of consciousness or headache are presented alone or in combination with other focal symptoms. However, studies suggest that this partly could be related to the higher frequency of subarachnoid haemorrhage as a cause of stroke in women.^28^ In our study, only four patients had isolated change or loss of consciousness. Healthcare providers should be aware of these potential sex differences in stroke presentation, but there is a need for more extensive studies presenting data on non-focal combinations of symptoms stratified by sex.

Only 171 (3.4%) of the included patients were between 18 and 44 years old; thus, our findings in this group must be considered cautiously. As seen in the general stroke population, also in the youngest, moderate and large strokes were covered by the FAST algorithm. In a study looking at patients from 18 to 55 years, the findings were similar, and FAST was considered useful also in this patient group.^24^ In the group of young women with mild strokes, only 23% had FAST symptoms at admission. Studies have shown that young patients who had a stroke are more frequently misdiagnosed, with misinterpretations of headache and peripheral vertigo as the most common diagnostic errors,^29^ and this may be more likely in women.^13^

Adding new symptoms to well-known public health campaigns could impact their effectiveness. This is because including additional symptoms might reduce the specificity of these campaigns and complicate easily remembered acronyms like FAST. Studies using the FAST acronym have demonstrated greater effectiveness in public education compared with those that did not incorporate it.^30^ In our exploratory analysis, we employed regression models to assess whether integrating new items from the NIHSS into the FAST algorithm could enhance outcome prediction. The outcome measure in our study was the mRS at 3 months. Our findings revealed that adding NIHSS items such as ‘visual field’ or ‘sensory’ to the FAST algorithm slightly increased the proportion of the patients with neurological impairments. Nonetheless, this enhancement did not significantly improve the predictive values in our models. A potential explanation for this could be the dichotomisation of mRS scores greater than 2, which may not be sufficiently sensitive to detect minor differences in outcomes. Furthermore, patients presenting with isolated impairments in visual field or sensory abilities generally experience milder strokes and, thus, have better functional outcomes. It is important to note that in our analysis, NIHSS items like ’sensory’ and ‘visual field’ served as markers for vision or sensory deficits. However, in the context of a public health campaign, conducting assessments as detailed as those in the NIHSS manual may not be feasible.^18^

This study has some limitations. First, the lack of reported non-focal symptoms like headache, vertigo, unsteady gait and dizziness is common in minor strokes and women. Thus, the non-presence of sex and age differences is limited to the FAST algorithm. Second, missing data on some NIHSS items were frequent, and some symptoms might be under-reported. As acute stroke treatment is highly time-dependent, the scoring of NIHSS in clear clinical cases is not always a priority, and especially items that require cooperation, like gaze and neglect, are missing in our data. Third, to increase our population with outcomes at 3 months, we transformed mRS from self-reported outcomes. This algorithm does not distinguish mRS 0, 1 or 2, and a good outcome will include patients with some disabilities. This limits our possibility of capturing small but relevant contributions to the outcome. Last, the FAST algorithm is used prehospital to improve symptom recognition in acute stroke and is not intended for prognostics. Thus, the calculated FAST from NIHSS at admission might differ from the prehospital score, as symptoms could change, limiting the generalisability.

Overall, 60% had at least one FAST symptom at admission, only 30% in mild strokes, with no sex or age difference. Adding new items from the NIHSS to the modified FAST algorithms led to a slight increase in strokes captured by the algorithms. Healthcare personnel and the public need to be aware of the low FAST algorithm precision in minor strokes, and our findings emphasise the importance of potential stroke symptoms knowledge and public education.

## Supplementary material

10.1136/bmjno-2023-000574 online supplemental file 1

## Data Availability

Data are available upon reasonable request.

## References

[1] Nor AM, McAllister C, Louw SJ (2004). Agreement between ambulance paramedic- and physician-recorded neurological signs with face arm speech test (FAST) in acute stroke patients. Stroke.

[2] Kleindorfer DO, Miller R, Moomaw CJ (2007). Designing a message for public education regarding stroke: does FAST capture enough stroke?. Stroke.

[3] Bray JE, Mosley I, Bailey M (2011). Stroke public awareness campaigns have increased ambulance dispatches for stroke in Melbourne, Australia. Stroke.

[4] Flynn D, Ford GA, Rodgers H (2014). A time series evaluation of the FAST national stroke awareness campaign in England. PLoS ONE.

[5] Wolters FJ, Paul NLM, Li L (2015). Sustained impact of UK FAST-test public education on response to stroke: a population-based time-series study. Int J Stroke.

[6] Wolters FJ, Li L, Gutnikov SA (2018). Medical attention seeking after transient ischemic attack and minor stroke before and after the UK face, arm, speech, time (FAST) public education campaign: results from the Oxford vascular study. JAMA Neurol.

[7] Toyoda K, Yoshimura S, Nakai M (2022). Twenty-year change in severity and outcome of ischemic and hemorrhagic strokes. JAMA Neurol.

[8] Krishnamurthi RV, Moran AE, Feigin VL (2015). Stroke prevalence, mortality and disability-adjusted life years in adults aged 20-64 years in 1990-2013: data from the Global Burden of Disease 2013 study. Neuroepidemiology.

[9] Rexrode KM, Madsen TE, Yu AYX (2022). The impact of sex and gender on stroke. Circ Res.

[10] Reeves MJ, Bushnell CD, Howard G (2008). Sex differences in stroke: epidemiology, clinical presentation, medical care, and outcomes. Lancet Neurol.

[11] Wang X, Phan HT, Li J (2020). Sex differences in disease profiles, management, and outcomes among people with atrial fibrillation after ischemic stroke: aggregated and individual participant data meta-analyses. Womens Health Rep (New Rochelle).

[12] Wang X, Carcel C, Hsu B (2022). Differences in the pre-hospital management of women and men with stroke by emergency medical services in New South Wales. Med J Aust.

[13] Yu AYX, Penn AM, Lesperance ML (2019). Sex differences in presentation and outcome after an acute transient or minor neurologic event. JAMA Neurol.

[14] Ali M, van Os HJA, van der Weerd N (2022). Sex differences in presentation of stroke: a systematic review and meta-analysis. Stroke.

[15] Shajahan S, Sun L, Harris K (2023). Sex differences in the symptom presentation of stroke: a systematic review and meta-analysis. Int J Stroke.

[16] Newman-Toker DE, Moy E, Valente E (2014). Missed diagnosis of stroke in the emergency department: a cross-sectional analysis of a large population-based sample. Diagnosis (Berl).

[17] Brott T, Adams HP, Olinger CP (1989). Measurements of acute cerebral infarction: a clinical examination scale. Stroke.

[18] Goldstein LB, Bertels C, Davis JN (1989). Interrater reliability of the NIH stroke scale. Arch Neurol.

[19] Sand KM, Wilhelmsen G, Naess H (2016). Vision problems in ischaemic stroke patients: effects on life quality and disability. Eur J Neurol.

[20] Johnson N, Nisar T, Criswell A (2022). Long-term disability outcomes for patients with ischemic stroke presenting with visual deficits. J Neuroophthalmol.

[21] Asplund K, Hulter Åsberg K, Appelros P (2011). The Riks-stroke story: building a sustainable national register for quality assessment of stroke care. Int J Stroke.

[22] Kasner SE, Cucchiara BL, McGarvey ML (2003). Modified national institutes of health stroke scale can be estimated from medical records. Stroke.

[23] Abzhandadze T, Reinholdsson M, Palstam A (2020). Transforming self-reported outcomes from a stroke register to the modified Rankin scale: a cross-sectional, explorative study. Sci Rep.

[24] Kaps M, Grittner U, Jungehülsing G (2014). Clinical signs in young patients with stroke related to FAST: results of the sifap1 study. BMJ Open.

[25] Arling G, Mazighi M (2021). Cost-effectiveness of mechanical thrombectomy for treatment of stroke: from modeling to real-world implementation. Stroke.

[26] Saber Tehrani AS, Kattah JC, Kerber KA (2018). Diagnosing stroke in acute dizziness and vertigo: pitfalls and pearls. Stroke.

[27] Di Carlo A, Lamassa M, Baldereschi M (2003). Sex differences in the clinical presentation, resource use, and 3-month outcome of acute stroke in Europe: data from a multicenter multinational hospital-based Registry. Stroke.

[28] Kapral MK, Fang J, Hill MD (2005). Sex differences in stroke care and outcomes: results from the Registry of the Canadian stroke network. Stroke.

[29] León Cejas L, Mazziotti J, Zinnerman A (2019). Misdiagnosis of acute ischemic stroke in young patients. Medicina (B Aires).

[30] Tan J, Ramazanu S, Liaw SY (2022). Effectiveness of public education campaigns for stroke symptom recognition and response in non-elderly adults: a systematic review and meta-analysis. J Stroke Cerebrovasc Dis.

