# Supplementary material for: The precision by the Face Arm Speech Time (FAST) algorithm in stroke capture, sex and age differences: a stroke registry study
Source: BMJ Neurol Open. 2024 Apr 15;6(1):e000574. doi: 10.1136/bmjno-2023-000574 (PMC11029396; doi:10.1136/bmjno-2023-000574)
Supplement: online supplemental file 1 [file bmjno-2023-000574supp001.pdf]

Supplementary table 1: Patients not included

**Total**		Included 5022	Not included 1341	P-Value
**Sex, n** (%)				<0.01
Women		2710 (46.0)	639 (47.7)	
Men		2312 (54.0)	702 (52.3)	
**Age**	Mean (SD)	73.6 (13.6)	75.5 (14.1)	<0.01
	Age groups, n (%)			<0.01
	18-44	171 (3.4)	50 (3.7)	
	45-64	962 (19.2)	195 (14.5)	
	65-79	2005 (39.9)	464 (34.6)	
	>79	1884 (37.5)	632 (47.1)	
**Stroke type** (%)				0.76
	Ischemic	4585 (91.2)	1031 (76.9)	
	Hemorrhagic	432 (8.6)	311 (23.2)	
**NIHSS**	Mean (SD)	5 (7)	11 (8)	<0.001
**Stroke severity** (%)[2]				<0.001
	Mild (NIHSS≤3)	2587 (51.5)	53 (27.3)	
	Mild to moderate	1364 (27.2)	69 (35.6)	
	Severe (NIHSS>15)	480 (9.6)	72 (37.1)	
**Died before 3 months follow-up**		538 (10.7)	430 (32.1)	<0.001
**Prior stroke**		768 (15.2)	258 (19.2)	<0.001
**FAST positive** (%)		2972 (59.2)	3 (0.002)	<0.001

NIHSS: National Institutes of Health Stroke Scale; FAST positive: including patients with one or more of the NHISS items facial palsy, motor arm, best language, and dysarthria.

Supplementary figure 1: Percentage of 5022 stroke cases covered when including new sub-items from the NIHSS to the FAST algorithm by sex.

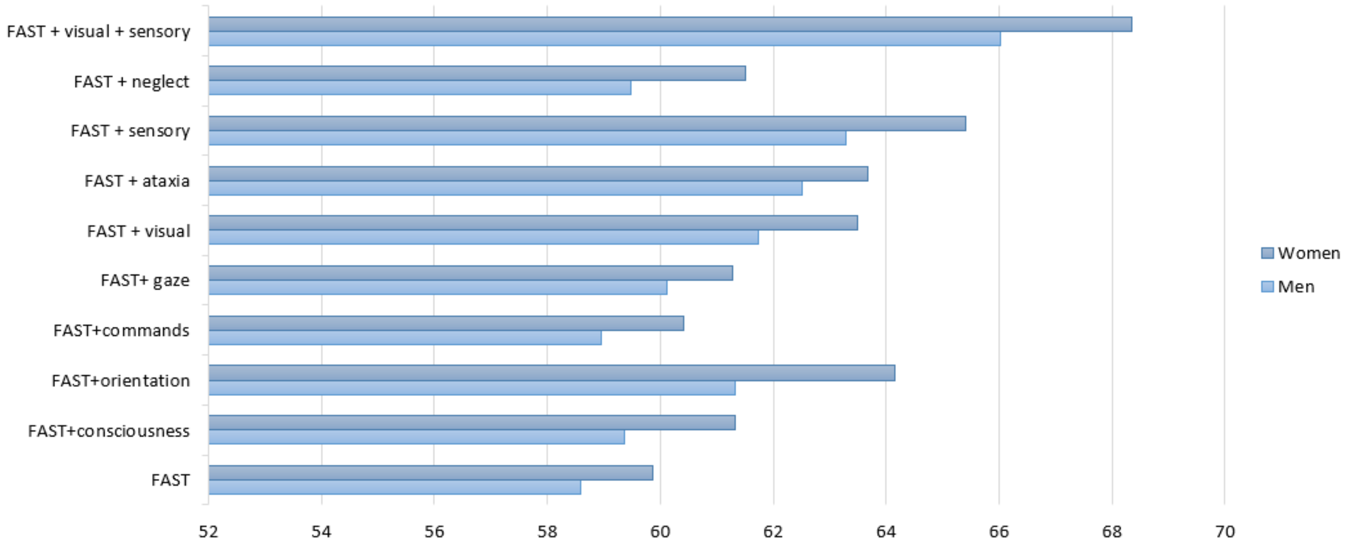

Supplementary table 2: Frequencies of isolated NIHSS items by sex

NIHSS sub item	Men	Women	n (%)
1a Consciousness	3	1	4 (0.1)
1b Orientation	28	35	60 (1.2)
1c Commands	0	1	1 (0)
2 Gaze	14	6	20 (0.4)
3 Visual	40	30	70 (1.4)
4 Facial Palsy	61	46	107 (2.1)
5 Motor Arm			
Right	5	5	10 (0.2)
Left	15	10	25 (0.5)
6 Motor Leg			
Right	5	14	19 (0.2)
Left	14	14	28 (0.6)
7 Ataxia	42	26	68 (1.4)
8 Sensory	64	63	127 (2.5)
9 Best Language	31	27	58 (1.5)
10 Dysarthria	44	36	80 (1.6)
11 Neglect	2	9	11 (0.2)

NIHSS=National Institutes of Health Stroke Scale

Supplementary table 3: Frequencies of the FAST algorithms by sex

N (%)	Total	Men	Women	P
FAST	2972 (59.2)	1588 (58.6)	1384 (59.9)	0.23
FAST-V	3141 (62.5)	1673 (61.7)	1468 (63.5)	0.12
FAST-S	3227 (64.3)	1715 (63.3)	1512 (65.4)	0.06
FAST-VS	3369 (67.1)	1789 (66.0)	1580 (68.3)	0.04

FAST: Face Arm Speak Time; FAST-V; Face Arm Speak Time Visual field; FAST-S: Face Arm Speak Time Sensory; FAST-VS: Face Arm Speak Time Visual field Sensory.
